# Chats about Medical Museums

**Published:** 1887-05-21

**Authors:** 


### Chats about Medical Museums.

By One of the Crowd.

(*Continued from p. 91, vol. ii.*)

### V.—Guy's Hospital.

In one of the two museums at Guy's Hospital is a little glazed frame or box, in which are contained some scraps of rusty iron. They are associated in a very intimate degree with the personal history of one John Cummings, an American sailor, who at the close of the last century, and at the age of about twenty-three, went ashore on the coast of France with some of his shipmates. They went to a fair, where they saw a mountebank amusing a company by swallowing pocket-knives. They were simple souls, those sailors, Yankees though they were, and never a doubt had they, apparently, that the mountebank's performance was a genuine one. When they got back to their ship Cummings seems to have pooh-poohed the achievement of the Frenchman, and vowed he could do the same if he liked; whereupon his shipmates dared him to try. Not liking to back out of it after his boasting, and "having a good supply of grog inwardly," he at length took out his own pocket-knife, and slipped it down his throat with the greatest ease. Partly by its own weight, and partly by a little hydrostatic pressure, which Cummings naturally enough supplied by more grog, the knife made its way down into the stomach, and no particular uneasiness was experienced. His shipmates, of course, applauded very heartily, and invited him to swallow another. Proud of their admiration, and emboldened by apparent impunity, the jolly tar declared that he was ready to swallow all the knives on board ship, and three more were instantly produced; and it is stated that he actually bolted them all. Three of them went in the ordinary course of nature, but one of the four he seems permanently to have appropriated. For six years he made no more experiments of the kind, but later on he repeated them frequently with the utmost unconcern. At length some little dyspeptic trouble appears to have overtaken him, and he had to seek medical advice. The doctors at first refused to believe his story, and looked upon him as a monomaniac. When they were induced seriously to examine him, they clearly detected a metal point projecting through the walls of the stomach. His state was at once pronounced hopeless, and he died; and from his ill-used stomach were removed the forty or fifty scraps of metal displayed here at Guy's. There is a small medal, the handle of a knife, which to a great extent seems to have resisted the corrosive action of the gastric juices, and the rest are blades and other portions of the articles swallowed, all of them more or less eaten away by the splendid digestive powers of this reckless idiot.

As it has been said, the collection at Guy's is disposed in two separate museums, both of which comprise models which might make a world-wide reputation for any institution. In the north wing of the new building are two rooms, chiefly occupied by upwards of two thousand specimens illustrative of the anatomy of the lower animals. This department, it is said, has been doubled during the past five years, and is continually enlarging. Among the most interesting of the objects in this section of the museum is a series of wax-models showing the changes in the egg during incubation, and another series in which one may trace the evolution of the brain in vertebrate animals from its most rudimentary stages up to the fully-developed human brain. But the most striking of the contents of this museum in the new building are some of the most marvellous models of healthy human anatomy that a combination of exceptional genius ever produced. The two men whose united talents have so enriched this hospital were the late Mr. Hilton, whose dissections cannot but strike even the unprofessional mind as masterly in the extreme, and the late Mr. Joseph Tawne, who modelled the objects thus dissected with a skill and a patient laboriousness simply amazing. This singular genius came up to London at the age of eighteen, bringing with him a small model of a skeleton which he himself had executed. It was shown to Sir Astley Cooper, who instantly recognised a work of consummate skill, and who pronounced upon it in terms of such glowing admiration, that the youth was at once engaged as modeller to the hospital, a post which he held for fifty-three years. It was a great stroke of fortune for Guy's that they should have secured this remarkable genius, whose labours have given them a series of anatomical and pathological models unrivalled by the most famous collections in the world.

Outside the medical profession little is known of this collection, and for the unscientific visitor who is not prepared for it, it affords rather a shock to the sensibilities to step, on a dull gloomy afternoon, into a long gallery in which are objects which it is impossible to distinguish from human trunks and heads and limbs laid out on tables just as they would appear after an exceedingly clever anatomist had laid bare their internal mechanism. Most persons unaccustomed to that kind of thing would probably experience some momentary shock; but most persons of ordinary intelligence would also be surprised to find how soon the thrill of aversion gives place to the keenest interest in the anatomical marvels displayed, and amazement at the almost equally marvellous artificial representations of them. On one table the visitor may study the minutest detail of the human arm; on another the muscles of the back are revealed, and on a third is a leg in which the ramifications of the veins and arteries of a well-formed leg are delineated in a manner which does not at all suggest the idea of a wax model, but that of an actual leg from which the skin has been deftly removed. The structure of the eye, the ear, the foot, the hand, is shown in objects all sense of repulsion to which is speedily lost in amazement at the infinite intricacy of the human form and the consummate skill of the artist. These models are really very wonderful, and they are said by competent anatomists to be as remarkable for absolute accuracy of detail as they are for general fidelity to nature.

The same genius has enriched the museum lying on the opposite side the avenue, running down from the front of the hospital in St. Thomas's Street to the back gates in King Street. In this museum also the wax models are quite startling in their vivid rendering of nature, and though there are hundreds of them, they are all by the same indefatigable genius, whose few simple implements, by the way, are exhibited in the anatomical museum, under the glass case containing the model skeleton which won for young Tawne his appointment at Guy's. The objects in this second museum are, as works of art, quite as astonishing as are the others; but they are altogether too painful to warrant any attempt to describe them. It is a very extensive pathological collection, exhibiting every phase and form of disease that has been met with in the hospital during the fifty-three years of the busy life of the modeller, and it need hardly be said that it is a collection which, to the medical man, is invaluable for study and reference, but which to the non-professional visitor is a collection to shudder over and forget—if he can.

(*To be continued.*)

